# STEPS – a patient centric and low-cost solution to ensure standards of TB care to patients reaching private sector in India

**DOI:** 10.1186/s12913-021-07342-y

**Published:** 2022-01-02

**Authors:** P. S. Rakesh, Shibu Balakrishnan, M. Sunilkumar, K. G. Alexander, Shibu Vijayan, Venkatesh Roddawar, P. P. Pramod Kumar, Jyothi Kailash, Akhilesh Kunoor, Midhun Rajiv, Anoop John, Rakesh Ramachandran

**Affiliations:** 1 WHO NTEP Technical Support Network, Kerala, India; 2 State TB Officer, Kerala, India; 3 Private Hospital Consortium for TB Free Kozhikode, Kerala, India; 4 PATH, New Delhi, India; 5 John Snow India Private Limited, New Delhi, India; 6 District TB Centre, National TB Elimination Program, Kozhikode, Kerala, India; 7 Coalition of Medical Professional Association for TB Free Ernakulam, Kerala, India; 8 Project JEET, Centre for Health Research and Innovation, Kerala, India

## Abstract

**Background:**

More than half of the TB patients in India seek care from the private sector. Two decades of attempts by the National TB Program to improve collaboration between the public and private sectors have not worked except in a few innovative pilots. The System for TB Elimination in Private Sector (STEPS) evolved in 2019 as a solution to ensure standards of TB care to every patient reaching the private sector. We formally evaluated the STEPS to judge the success of the model in achieving its outcomes and to inform decisions about scaling up of the model to other parts of the country.

**Methods:**

An evaluation team was constituted involving all relevant stakeholders. A logic framework for the STEPS model was developed. The evaluation focused on (i) processes - whether the activities are taking place as intended and (ii) proximal outcomes - improvements in quality of care and strengthening of TB surveillance system. We (i) visited 30 randomly selected STEPS centres for assessing infrastructure and process using a checklist, (ii) validated the patient data with management information system of National TB Elimination Program (NTEP) by telephonic interview of 57 TB patients (iii) analysed the quality of patient care indicators over 3 years from the management information system (iv) conducted in-depth interviews (IDI) with 33 beneficiaries and stakeholders to understand their satisfaction and perceived benefits of STEPS and (v) performed cost analysis for the intervention from the perspective of NTEP, private hospital and patients.

**Results:**

Evaluation revealed that STEPS is an acceptable model to all stakeholders. IDIs revealed that all patients were satisfied about the services received. Data in management information system of NTEP were consistent with the hospital records and with the information provided by the patient. Quality of TB care indicators for patients diagnosed in private hospitals showed improvements over years as proportion of TB patients notified from private sector with a microbiological confirmation of diagnosis improved from 25% in 2018 to 38% in 2020 and the documented treatment success rate increased from 33% (2018 cohort) to 88% (2019 cohort). Total additional programmatic cost (deducting cost for patient entitlements) per additional patient with successful treatment outcome was estimated to be 67 USD. Total additional expense/business loss for implementing STEPS for the hospital diagnosing 100 TB patients in a year was estimated to be 573 USD while additional minimum returns for the hospital was estimated to be 1145 USD.

**Conclusion:**

Evaluation confirmed that STEPS is a low cost and patient-centric strategy. STEPS successfully addressed the gaps in the quality of care for patients seeking care in the private sector and ensured that services are aligned with the standards of TB care. STEPS could be scaled up to similar settings.

## Background

India has highest number of TB and Drug resistant TB cases in the world [1]. More than half of the TB patients in India seek care from the private sector [2]. There were concerns about the unknown quality of TB care in private sector, lack of systems for treatment adherence support and a high loss to follow-up that could increase the risk of drug resistance [3, 4].

National TB Elimination Program (NTEP) have recognized that effective engagement of the private sector on a scale commensurate with their dominant presence in Indian healthcare is crucial to achieve Universal Access to TB Care. Two decades of attempts to improve collaboration between the public and private sectors, have not worked in India except in a few innovative pilots [5]. Many pilot models to strengthen public private partnerships for TB control, could not be scaled up due to lack of addressing the concerns of private sector and heavy dependency on the public sector [6, 7]. Recent models such as Patna and Mumbai models which had addressed the concerns of private sector had heavy dependency on intermediary agency and relied heavily on donor fundings [8, 9]. Such models did not expand once the projects ended. Domestic investments have facilitated private sector engagements but have not expanded to scale. Most of the NTEP’s direct engagement models focused only on improving TB notifications and tasks beyond notification have gaps in performance [10].The System for TB Elimination in Private Sector (STEPS) is a model evolved as a solution to address gaps in the quality of TB care in the private sector. STEPS is envisioned as an equal partnership between the public and private sector for the benefit of society with TB elimination as the outcome. Concept and evolution of STEPS, challenges in implementation and early outcomes were documented [11].STEPS has three components: (i) a private hospitals TB consortium (PHOTON), (ii) a coalition of medical professional associations (COMPAcT) at state and district levels, and (iii) a STEPS center in each private hospital. STEPS center within a hospital is a single window for diagnostic and treatment services, notification, patient linkage with social welfare, contact investigation, chemoprophylaxis and treatment adherence support. A central person (STEPS lead) nominated by the hospital management, work together with contact persons (STEPS links) for each in-house department in a hub-and-spoke model. The STEPS lead and links are typically staff nurses. STEPS Links from various in-house clinical departments transfer the patients and related information to the STEPS Lead. Patients visit STEPS center where education, counselling, support and linkages for molecular diagnostics, anti TB treatment initiation, contact investigations, chemoprophylaxis, social welfare schemes and air borne infection control are provided. STEPS Lead follows up the patient periodically over telephone and provides treatment adherence support, monitors adverse drug reactions, reminds clinical follow up and schedules reviews. Patient visits the concerned in house departments for clinical follow up. STEPS Lead enters information in NIKSHAY- the case based digital surveillance system of National TB Elimination Program (NTEP) [12].

STEPS was implemented in all 14 districts of Kerala state, India since January 2019. All 14 districts formed PHOTON for policy support and COMPAcT for advocacy with doctors. Of the 446 hospitals mapped, 318 established STEPS centers during 2019 and the remaining in 2020. Project JEET, a Global Fund supported patient-provider support agency, rendered services of a state lead and five city officers for 2 years to assist NTEP in establishing STEPS.

No formal evaluation of STEPS was done till date. A joint monitoring mission (JMM) held in India in November 2019 led by the World Health Organization and global developmental partners, including 165 multidisciplinary experts recommended to conduct a formal evaluation of STEPS to inform its expansion to other states [10]. Based on the recommendations of JMM, we evaluated the STEPS model to judge the success of the model in achieving its proximal outcomes and to inform decisions about scaling up of the model to other parts of the country. The evaluation focused on (i) processes such as whether the activities have taken place as intended and (ii) proximal outcomes such as improvements in quality of care and strengthening of TB surveillance system. A cost analysis was also performed to inform decision for scale up.

## Methodology

**Fig. 1:**
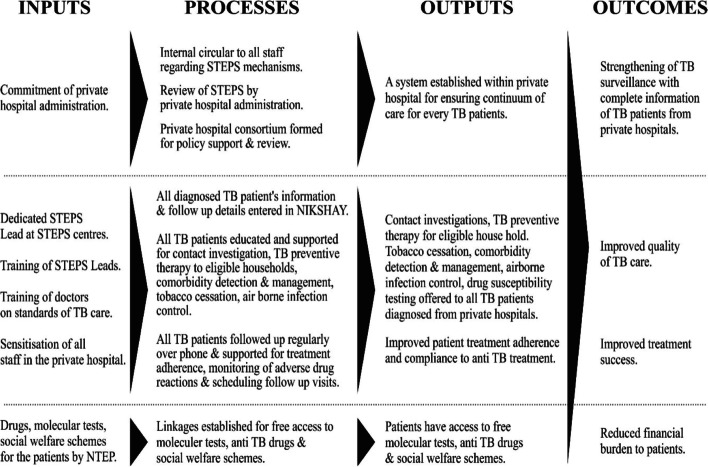
Logic Framework for STEPS

We developed the evaluation based on the framework for program evaluation in public health by Center for Disease Control and adhering to the evaluation standards set by the Joint committee on standard for educational evaluation, USA [13, 14]. An evaluation team was constituted involving all relevant stakeholders. A logic framework for the STEPS model was developed as shown in Fig. 1.

Evaluation was designed in five steps

1. Facility Visit to STEPS centres for assessing infrastructure and process
2. Validation of the patient data in NIKSHAY by telephonic interview of TB patients availing services from STEPS centres
3. Analysis of quality of patient care indicators over 3 years from NIKSHAY
4. In-depth interviews (IDI) with beneficiaries and stakeholders
5. Cost analysis from the perspective of NTEP, private hospital and patients

The evaluation protocol and tools were prepared based on group consensus by the evaluation team and was finalized with suggestions from two district program managers, two doctors from private sector, two STEPS Leads, state chair of coalition of professional medical association and experts from Central TB Division and WHO India office. Sample size was arbitrarily fixed based on group consensus; operational and logistic feasibility was also given a higher weightage.

### Step 1. Facility visit to STEPS centres for assessing infrastructure and process

Thirty (30) STEPS centres out of 318 were selected using simple random sampling for physical assessment with a 15-point checklist. Checklist included points (shown in Table 1) to assess the infrastructure and functionality of the STEPS centres. Eighteen (18) were visited during January–March 2020 and the remaining were visited during November 2020–January 2021due to the travel restrictions imposed by unprecedented situation of COVID-19. Data obtained were presented as frequencies and percentages.

**Table 1:** Assessment of infrastructure and functionality of STEPS Centers (*N* = 30)

Checklist	Number (%)
Presence of dedicated STEPS Lead for the hospital	30 (100%)
STEPS Lead trained in STCI & NTEP	29 (96.6%)
Training for doctors on STCI conducted	28 (93.3%)
An internal circular to all hospital staff by Administration regarding STEPS	22 (73.3%)
Details of STEPS services displayed for public	17 (56.6%)
Receptionist/Patient Help Desk aware of STEPS centre	27 (90%)
Access to Molecular testing for TB available	26 (86.7%)
STEPS Lead demonstrated process of ensuring treatment support to patients with the help of documents	26 (86.7%)
STEPS Lead explained patient data using NIKSHAY	29 (96.7%)
Airborne Infection Control Kit for issuing to patients available	23 (76.7%)
NTEP drugs stock matching with stock register (*N* = 14)	14/14 (100%) *
Cough hygiene IECs displayed at patient waiting areas	24 (80%)
Review of TB activities of STEPS centre by Hospital Administration	19 (63.3%)
System for notification of all cases in NIKSHAY with triangulation of clinical data with pharmacy data, lab data	17 (56.7%)
Participated in quarterly review of STEPS by district private hospital consortium (PHOTON)	24 (80%)

*14 out of 30 hospitals were stocking NTEP drugs, 9 were getting the drugs as and when needed from NTEP because of low case load and 7 hospitals were prescribing only private anti TB drugs

### Step 2. Validation of the patient data in NIKSHAY by telephonic interview of TB patients availing services from STEPS centres

Two patients were selected from each of the 30 STEPS centres by simple random sampling from the NIKSHAY notification register for 2019 and 2020. They were contacted over telephone and enquired about seven points including date and basis of diagnosis, date of treatment initiation, HIV status, date of universal drug susceptibility testing, direct benefit transfer and treatment outcome. Three of them could not be contacted (one hospital had only one patient notified, one patient did not consent and one was not picking phone despite repeated attempts). Available hospital records of those patients were also verified. The information provided by the patients and obtained from hospital records were compared with the data entered in NIKSHAY to assess concordance. Concordant data in each selected data points were expressed as frequency and percentage.

### Step 3. Analysis of quality of patient care indicators

Quality of patient care indicators based on Standards of TB Care in India (STCI) were captured from NIKSHAY for the years 2018, 2019 and 2020. Indicators included proportion of patients notified by private sector (a) with a microbiological confirmation, (b) offered drug susceptibility testing at baseline, (c) knew their HIV status, (d) received direct benefit transfer, (e) with reported treatment outcome and (f) with successful treatment outcome.

Proportion of private sector patients tested using public GeneXpert was obtained from monthly reports of GeneXpert sites. The data obtained were presented as frequencies and percentages and comparison was made across the years. Report by Government of Kerala regarding yearly analysis of private anti TB drugs sales was obtained and studied [15].

### Step 4. In-depth interviews with beneficiaries and stakeholders

In-depth interviews were conducted with patients, hospital administrators, staff nurses (STEPS Leads), private doctors, NTEP program managers at district and sub-district levels and leaders of professional medical associations. Patients to be interviewed were identified by the evaluation team during the initial conversation with patients during step 2, based on their willingness to talk, clarity in communication and ability to access online meeting platforms. Nurses, doctors and hospital administrators (maximum of one person in this category per hospital) were identified during facility visit in step 1 based on their willingness to spend time. District/sub-district NTEP program managers who had more than 3 years of experience and working in areas with numerous private hospitals were nominated by state NTEP program manager. Members of professional medical associations who work closely with TB and private sector for long time were nominated by the chair of COMPAcT. IDI were conducted till saturation was reached and no new themes arose among each category of informants. Conscious efforts were taken to ensure geographical representation by giving preference in the order of the interviews to participants from different districts.

Interviews with hospital administrators, private sector doctors and staff nurses were conducted face to face during the visit to hospitals by prior appointments. However due to travel restrictions because of COVID-19 pandemic, interview of patients, NTEP program managers and professional association leaders were conducted through online platforms. Participants were approached over telephone, purpose of interviews were communicated and the prerequisites during the interview (stable internet connection, peaceful atmosphere and preferably with video turned on) were communicated.

A guide for interview was developed. Time was fixed based on the convenience of participant. Other than the participant and interviewer, one more team member was present during the interview recording the proceedings and monitoring verbal and nonverbal interactions. The aims of the study and implication for participation were explained to all participants at the beginning of the interview. Informed written consent and permissions for recording was obtained from the participants prior to the interviews. Confidentiality was ensured and participants were informed of the chance to opt out at any stage without stating reason. All but one NTEP program manager and one patient contacted could participate. IDIs were conducted with 8 patients, 4 hospital administrators, 5 staff nurses, 6 private sector doctors, 6 NTEP program managers at district and sub-district level and 4 professional medical association leaders.

Major themes covered during the interview were (a) experiences with STEPS (b) relationship with partners (c) challenges faced during implementation and (d) suggestions for improvement. All but three interviews were conducted in the local language Malayalam. Twenty-three IDIs were conducted by A1 (male, public health expert) and 10 by A2 (male, health system expert); both of them were well experienced in conducting qualitative studies and were fluent in local language. Online interviews were through Zoom (Zoom Video Communications, Inc.). The interviewers ensured that the themes were fully discussed and that all participants were given a chance to express their views fully. One patient and two STEPS Leads rang back and discussed additional points after formal interviews. Each interview lasted for approximately 30 min [Range 18 min–50 min].

IDI were later transcribed verbatim and translated into English. For online interviews, one team member recorded the proceedings identifying key themes and monitoring verbal and nonverbal interactions by watching the video recordings. Detailed field notes taken during the interviews were verified against recordings by an investigator who did not compile the original notation. The transcripts were then manually coded by researchers (MR/RR/AJ) and emerging themes and sub themes were identified. Coding was then verified by another investigator (PSR/SB) on a later date. Sections with similar coding were grouped according to the predetermined themes. Repeated themes were marked as important in red font colour. All the flagged statements were put together and synthesized. The team read the transcripts and notes and reached a consensus. Any disagreements were discussed regularly within the team to reach a consensus regarding theme coding. Important quotations were quoted which evoked spontaneous discussion, around which a lot of time was spent and had some emotional cues attached with.

### Step 5. Cost analysis from the perspective of NTEP, private hospitals and patients

We conducted a retrospective activity-based costing analysis. Information sourced from state and district NTEP financé team for identifying major cost drivers of STEPS. We collected data on actual costs incurred for various components such as salaries and field expenses of project JEET staff, trainings and sensitisation for STEPS, actual expenses for tests, drugs, air borne infection control kits and direct benefit transfers, programmatic cost to establish and maintain specimen collection and transportation system connecting private hospitals and organising meetings of PHOTONs and COMPAcTs. Since the incentive to the private hospitals were not fully paid by NTEP, based on additional number of patients notified and with reported outcomes, incentive for notification to private hospitals and incentive for providing outcome to private hospitals were estimated assuming full payment based on eligibility. Additional expenses on supervision, monitoring & evaluation, printing of directory, patient information booklets, IEC materials and supply chain management were also derived in consultation with NTEP program managers based on actual statement of expenditures from all districts and their proportionate share for implementation of STEPS. We labelled the costs of services which directly goes to patient (GeneXpert cartridge, Chest Xray reimbursement, NTEP drugs, AIC Kit, Direct benefit transfer to patient) as cost for patient entitlements and the rest as programmatic costs.

We also interviewed hospital administrators, finance managers and staff nurses of two well-functioning STEPS centres in detail to understand their additional expenses in implementing STEPS centre. Interviews focussed on listing out all activities related to STEPS and identifying the cost involved from the hospital side for each activity. Human Resource cost was calculated based on the approximate time spent by staff for additional activities, their total working hours per months and average monthly salary. Expenses for telephone charges, loss due to forgone profit in TB drugs, loss due to forgone profit in molecular tests for baseline rifampicin testing was calculated for hospital, assuming all patients received full benefits. Based on the actual figures in improvement in patient notifications and treatment outcome rates from private sector in the state after implementation of STEPS, we estimated the additional expenses or business losses and additional returns to a private hospital diagnosing 100 TB patients in a year. Change in treatment success rates were estimated taking 70% as baseline from two previous studies (one unpublished) where a cohort of patients initiated on TB treatment from private hospitals were followed up to obtain treatment outcome in 2017 [16].

Average number of visits to the hospital by a patient before and after implementation of STEPS was also elicited by looking at hospital records. Average consultation fee per visit, cost for follow up investigations, treatment of co-morbidity for TB patients, cost for one course of private anti TB drugs and one GeneXpert test were obtained from the hospital. With the information, we tried to estimate the cost details for a TB patient who would have been Lost to Follow Up from a private hospital prior to STEPS implementation and now availed all services through STEPS. All costs were captured in Indian Rupee (INR) and converted to USD at a conversion rate of one INR = 0.013 USD.

Ethics approval was obtained from Independent Ethics Committee of Centre for Public Health Protection (IEC-CPHP-2019-11/12), Kerala, India.

## Results

Assessment of infrastructure and process of STEPS centres obtained through facility visits were shown in Table 1. All the hospitals visited had dedicated STEPS Leads. Hospital administration had issued formal communication regarding STEPS in 22(73.3%) hospitals. In 26 (86.7%) facilities, STEPS lead could demonstrate the process of ensuring treatment support to patients with the help of documents like registers and phone call logs. In 19 (63.3%) STEPS centres, hospital administration reviewed the activities of STEPS centre periodically. Seventeen (56.7%) hospitals demonstrated how they ensured 100% notification by triangulation of the information from medical records, pharmacy and laboratory.

Data in NIKSHAY were consistent with the information provided by the patient. Thirty-eight patients interviewed were eligible to have their treatment outcome reported. All those treatment outcomes were reported correctly in NIKSHAY. Details of validation of the patient data in NIKSHAY by actual telephonic interview of TB patients availing services from STEPS centers were shown in Table 2.

**Table 2:** Validation of the patient data in NIKSHAY by telephonic interview of TB patients availing services from STEPS centres (*N* = 57)

Data points	Information in NIKSHAY consistent with Patient Interviews & record verification
Date of TB diagnosis	56/57 (98.2%
Basis of TB diagnosis	54/57 (94.7%)
Date of treatment initiation	57/ 57 (100%)
HIV status	48 / 48 (100%)
Date of UDST	28/29 (96.5%)
DBT benefit received	31/31 (100%)
Outcome Reported	38/38 (100%)

Comparison of quality of patient care indicators for 2018, 2019 and 2020 were shown in Table 3. In 2019, 99% of the TB patients notified from private sector had their outcome reported with a treatment success rate of 88%. Number of specimens from private sector tested using public GeneXpert machines increased from 7606 in 2018 to 14,210 in 2019. Proportion of TB patients notified from private sector with a microbiological confirmation of diagnosis improved from 25% in 2018 to 38% in 2020 and those who knew their HIV status improved from 42 to 83%.

**Table 3:** Comparison of quality of patient care indicators (2018,2019, 2020)

Indicator	2018	2019	2020
Number of TB cases notified by the private sector	3981	5003	5795
Proportion of TB cases (out of total notified) notified from private sector	16.2%	19.6%	28.9%
Number (%) of microbiologically confirmed cases among notified TB	995 (25%)	1951 (39%)	2202 (38%)
Number (%) of notified patients offered Rifampicin drug resistance testing at baseline	637 (16%)	1951 (39%)	3187 (55%)
Number (%) of notified TB patients who know their HIV status	1672 (42%)	4102 (82%)	4809 (83%)
Number (%) of notified patients received direct benefit transfer	1273 (32%)	3552 (71%)	4056 (70%)
Number (%) of notified patients whose treatment outcome was reported in Nikshay	1393 (35%)	4952 (99%)	
Number (%) of notified patients who had successful treatment outcome reported in Nikshay	1324 (33%)	4402 (88%)	

Drug sales data showed a decline in the sale of anti-TB drugs from 1.6 million rifampicin units in 2018 to 0.87 and 0.5 million rifampicin units in 2019 and 2020 respectively.

### Insights from in-depth interviews

- Experience with STEPS

All the patients interviewed were satisfied with the services they received from private hospitals. What made them most happy is the calls they used to receive from the hospital enquiring their wellbeing. Patients conveyed that they felt protected and cared.

One of them said that he got a second opinion from another private hospital where he was suggested the same course of tests and treatment, so he preferred to continue his treatment with the first hospital. Another patient who was diagnosed at a tertiary care private hospital said that he was asked to continue his treatment from STEPS centre of their spoke hospital near to his home and was advised to visit main hospital once in 3 months only.

“*I have sought treatment for all my illnesses and my family members illness in many private hospitals. But it was for the first time I have seen a hospital calling us and enquiring our wellbeing regularly. I was surprised - how can a private sector hospital do this? I had the WhatsApp number of the sister (STEPS Lead). I used to send a message every day after taking my drugs. She also helped me scheduling my appointments. As soon as I reach hospital, I will go directly and meet sister. She will accompany me to doctor. Care from her was more than that provided by my relatives*”- a 46-year-old patient (Textile shop owner, 12^th^ standard)

“*My father had TB 5 years back. First time he took treatment for only two months. He again had TB. That time he was put on anti TB drugs again from a private hospital. He had abdominal pain and fatigue whenever he took drugs, so, he used to stop drugs in between and go to another hospital. He has gone to multiple private hospitals and clinics. Finally, he died. I had seen his sufferings and I was really afraid when I had TB. But the support I received from this hospital was beyond words. I am always grateful to X hospital. If this kind of facilities were there at that time, I feel my father would have been with me now*”- a 36-year-old patient (Clerk in a private company, Batchelor’s degree completed)

“*I feel this is a good initiative. Patients are benefitted for sure. It has increased our workload, but we feel it is benefiting our patients. Previously they were not receiving all these services. In fact, we did not know such things. We can do many more such things*.”- STEPS Lead (Female, Nurse,37 years)

Doctors working in private sector agreed that STEPS helped them in identifying what is happening to their patients and ensured access to high end investigations even for poor patients approaching them.

“*Earlier if you ask me how many TB patients I had and what is happening to my patients, I had no clue. But now I can confidently tell every detail about each of my patient*.”- private sector doctor (pulmonologist, 43 years)

“*Earlier I was scared to refer a patient to NTEP. I will not get any feedback and I will lose my patient. Patients whom I referred had many bad experiences. Now they (NTEP) provide everything for the patient here. It is such a wonderful system*.”- a private sector doctor (Pulmonologist, 52 years)

NTEP program managers and field staff were happy with the STEPS initiative. Field staff were happy about the system in private hospital. They agreed that collaboration with private sector and involvement of private doctors for TB Elimination has increased many folds after implementation of STEPS. They felt that, STEPS has also helped to ensure continuity of care to TB patients during COVID pandemic.

“*Private sector is now more active that public sector in TB Elimination activities. Private doctors who considered us as great enemies once, come forward and advocate for TB Elimination now. During COVID times, when some of our public facilities were converted as exclusive COVID hospitals, we asked many of our patients to get their consultations and tests from private hospitals through STEPS. They even waved off consultation fee for the patients whom we referred. It was because of STEPS, our TB notification has not gone down during COVID”-* District Program Manager, NTEP (Female 42 years).

Leaders of professional associations commended that STEPS is the right solutions that will accelerate the fight for TB Elimination. They also mentioned about the role of STEPS platform for facilitating better participation of private sector in fighting COVID-19

b) **Relationship with Partners**

Private administrators were happy about STEPS initiative and their relationship with their counterparts. They reiterated their willingness to fight for social causes.

“*My experiences with government were always been bad. Every time the person changes, their response also changes. I agreed for this as it was a ‘system’ and really, we could feel the difference. The quality of products eg air borne infection control kits were also good. We are willing to collaborate more with Government for such initiatives to help society*”- a private hospital owner (non-Doctor, 800 bedded hospital)

All STEPS Leads interviewed reported no issues with NTEP staff in obtaining guidance or services.

“*Mrs X (TB Health Visitor of NTEP) is very supportive. Whenever I have a doubt, I call her. There will be staff from NTEP in all areas. Mrs X will help me to link our patients with NTEP staff based on patient’s residential address. Those staff will help them receive additional benefits from Government and nutritional support kits. Mrs X also helped one of our patients to solve a family conflict with his son and daughter-in -law by visiting there*” – STEPS Lead (Female, Nurse, 49 year)

**“***STEPS is one of the best initiatives that I have seen in my overall career of 24 years in NTEP. We had so many issues with PPP. No doctor will hear us and were willing to see us previously. Now the communication is very smooth as we have contact person (STEPS Leads) in every hospital. We have a WhatsApp group also with all STEPS Leads. It has made our life so simple***” –** a senior NTEP key staff (Male, 54 years)

c) Facilitators.

Hospital administrators mentioned the change in attitude of the public sector as the biggest facilitator for STEPS establishment

“*Private sector is always willing to help Government in dealing with social issues like TB. We are even willing to forgo our profits for TB patients. Our only concern was the interference from external agencies into our day-to-day activities. It was such a radical change with STEPS. Government never burdened us and were sensitive to our concerns (patient confidentiality, fear of losing patients, too much documentation). After all, STEPS is for our own patients. Why is this not being scaled up to other cities in India?* “- a private hospital administrator (Medical doctor, 100 bedded hospital)

STEPS Leads reported that the biggest facilitator was the support from the side of hospital administration. Doctors agreed that involving staff nurse for co-ordination was a good move as it reduced their burden on documentation.

d) Challenges.

STEPS Leads, though felt that their work load improved a bit, were happy about STEPS initiative. They felt that the biggest challenge in STEPS implementation was constantly reminding all the staff within their hospital.

Doctors reported that many doctors in the hospital could not participate in trainings as there would never be a time convenient to all. Training the staff due to rapid turnover of paramedical staff at private hospitals was also reported as a challenge.

One field staff who had many private hospitals in his area, requested a separate system for supply chain management to supply drugs to private hospital.

“*Supplying drugs is also part of our job responsibility. In addition to 16 public hospitals, now I need to issue drugs to 13 STEPS centres also. I feel a bit overburdened. It would have been good if entire supply chain management of drugs is managed through some other mechanisms*” – Senior Treatment Supervisor, NTEP (52 years old, 21 years of experience)

Inability to cater to the needs of the individual practitioners through the existing system was quoted as a challenge by the professional medical association leader.

**e) Suggestions for Improvement**: Suggestions for further improvement emerged for the interviews were (1) establishing a support system for supply of drugs and other logistics from NTEP to private hospitals (2) developing a digital learning platform for trainings and updates based on convenience of the doctors and staff of private hospital and (3) improving current involvement of individual practitioners and hospitals through a hub and spoke model.

### Cost analysis

Details of cost for implementing STEPS from NTEP were provided in Table 4. Additional total cost from NTEP per additional patient with successful treatment outcome was estimated as 136 USD. Of this, 69 USD is the cost for ‘patient entitlements’ that directly goes to patients (GeneXpert cartridges, drugs, chest x-ray reimbursement, airborne infection control kit and direct benefit transfer). Total additional programmatic cost (deducting cost for patient entitlements) per additional patient with successful treatment outcome was estimated to be 67 USD. Of these, 33 USD per additional patient with successful treatment outcome was for the salary and field expenses of staff from project JEET.

**Table 4:** Cost for NTEP for implementation of STEPS

Cost Category	Description	Average cost for 12 months in USD
1. **Staff Cost**	Salary of PPM Lead and 5 city officers of Intermediary agency	49,000
	Field Expenses of PPM Lead and 5 city officers of Intermediary agency	31,000
2. **Training & Sensitisation**	Training, Retraining and Sensitisation of doctors and STEPS Leads	27,788
3. **Diagnosis**	Specimen pick up transportation from private hospitals to GeneXpert site	1669
	14 additional Lab Technician posted at GeneXpert site to handle extra specimens from private sector	30,333
	6604 GeneXpert cartridge for testing additional samples from private sector*	110,066
	10,000 Number of Chest X ray Reimbursed for diagnosing TB to private hospitals*	27,776
4. **Treatment**	2000 additional patients from private sector put on NTEP drugs*	28,694
	Air born Infection Control Kits provided*	5555
5. **Administrative Cost**	Meetings of Coalition and Consortiums	7778
	Additional expenses on Supervision, Monitoring & Evaluation	1388
	Printing of directory, Patient information booklets, IEC materials	6944
	Additional expense for supply chain management	10,933
6. **Incentive**	Additional Incentive for Notification to private hospitals	12,597
	Additional incentive for providing outcome to private hospitals	24,194
	Additional Incentive to the patient getting treatment from private sector	36,250
**Total Additional Cost from NTEP**		411,965
**Total Additional Cost (programmatic cost and cost for patient entitlements) per additional patient with successful treatment outcome**		136
**Additional Programmatic Cost (deducting cost for patient entitlements) per additional patient with successful treatment outcome**		67

Estimated cost sheet for a private hospital diagnosing 100 TB patients in a year for implementing STEPS is provided in Table 5. Total additional expense/business loss for implementing STEPS for the hospital was estimated to be 573 USD while additional returns for the hospital was estimated to be 1145 USD.

**Table 5:** Estimated Cost sheet for a private hospital diagnosing 100 TB patients in a year for implementing STEPS

Additional Expenses/ Business Loss to private hospital (USD)	Additional Returns to private hospitals (USD) per year^**a**^
Telephone charges = (0.02 USD per call *1000 calls) = 20USD	18 TB patients, who were previously lost to follow up, return to hospital/spoke centre for average of 5 additional visits (3 USD consultation fee & 3.5 USD for follow up investigations and treatment of co-morbidity * 18 * 5 visits) = 585 USD
Loss due to forgone profit in TB Drugs = (2.5USD *100) =250	
Loss due to forgone profit in Molecular tests for baseline Rifampicin testing (2USD*100) = 200 USD	Incentive for Providing Treatment Outcome (7 USD*60 additional outcome) = 420 USD
6000 Minutes of staff Nurse (12.5 working days) =103 USD	Incentive for TB Notification (7 USD*20 additional notification) = 140 USD
**Total additional expense/business loss: 573 USD**	**Total Additional Returns: 1145 USD**

^a^ Additional returns to hospitals through increased client load through referral of friends and relatives by loyal customers not calculated

Estimates of cost details for a TB patient who would have been Lost to Follow Up from a private hospital prior to STEPS implementation and now availed all services through STEPS have been shown in Table 6. Additional expenses for such a patient have been estimated to be 85USD while cost saving/ additional income for the patient was estimated to be 92 USD.

**Table 6:** Cost details for a TB patient who would have been Lost to Follow Up from a private hospital prior to STEPS implementation and now availed all services through STEPS

Additional Expenses for the Patient ^**a**^	Cost Saving/ Additional Income for the patient^**$**^
Additional 5 consultation at private hospital/ spoke centre (3USD for consultation fee, 10 USD for follow up investigation and treatment of co-morbidity, 4 USD for travel per visit to hospital/spoke canter) = 5*17 USD = 85 USD	TB Drugs - 17 USD Expense for GeneXpert - 28 USD Expense for X-ray − 03 USD AIC Kit - 02 USD DBT - 42 USD
**Total: 85 USD**	**Total: 92 USD**

^a^ Indirect cost other than travel not included, ^$^cost saving through improved health not estimated

## Discussion

Evaluation revealed that private hospitals following up their clients with TB for clinical and public health actions is feasible. We observed that that services through STEPS at private hospitals are aligned with the standards of TB care. STEPS was found as an acceptable model to all stakeholders. Patients were satisfied about the services received. Data in NIKSHAY were consistent with the hospital records and with the information provided by the patients. Quality of TB care indicators for patients diagnosed in private hospitals showed improvements over years, as proportion of TB patients notified from private sector with a microbiological confirmation of diagnosis improved from 25% in 2018 to 38% in 2020 and the documented treatment success rate increased from 33% (2018 cohort) to 88% (2019 cohort). Total additional programmatic cost (deducting cost for patient entitlements) per additional patient with successful treatment outcome was estimated to be 67 USD. Total additional expense/business loss for implementing STEPS for the hospital diagnosing 100 TB patients in a year was estimated to be 573 USD while additional returns for the hospital was estimated to be 1145 USD. Suggestions for further improvement of STEPS were (1) establishing a support system for supply of drugs and other logistics from NTEP to private hospitals (2) developing a digital learning platform for trainings and updates based on convenience of the doctors and staff of private hospital and (3) improving current involvement of individual practitioners and hospitals through a hub and spoke model.

Stakeholders reported that STEPS was helpful even during COVID-19 period, not only to ensure services for TB patients, but also facilitated partnerships for COVID-19 management. The number and proportion of TB notification from private sector and the quality indicators in the pandemic year revealed that STEPS had demonstrated good resilience during pandemic period. Separate studies are required to document how STEPS ensured this resilience and what are the benefits of STEPS beyond TB Elimination.

Evaluation revealed that STEPS is a ‘patient centric’ model where the patient can approach any provider according to his/her choice and get uniform high quality TB care. Through STEPS, private hospitals proactively supported patients to make decisions and participate in their own care. STEPS also improved health outcomes of patients. This is consistent with the NTEP’s vision that all patients should receive high standards of care from the providers of his/her choice.

Many private sector engagement models in India had heavy dependency on intermediary agencies for following up patients, making the establishment cost huge for scale up [8–10]. The efforts for establishing sustainable systems were minimal. In STEPS, the intermediary agency never attempted to contact any patients directly, rather they worked for establishing a system within private hospitals to ensure continuum of care for every TB patient. Form April 2021 onwards, support of intermediary agency has been withdrawn and the STEPS is running by itself. Though it might be too early to comment on the sustainability of STEPS in the absence of additional staff provided through project JEET, it could be undoubtedly stated that the efforts from the beginning were in the direction for establishing sustainable systems.

Three UATBC (Universal Access to TB care) pilots projects for private sector engagement were implemented in Patna, Mumbai and Mehsana in India [8].Average recurring cost per notified case was estimated to be between 90 USD and 100 USD for urban pilots in Mumbai and Patna and around 50 USD for rural pilots in Mehsana [17]. Total programmatic cost from NTEP in STEPS model was estimated to be 67 USD per additional patient with a successful outcome. It has to be noted that we calculated total programmatic cost per additional patient with a successful treatment outcome. STEPS appeared to be a low-cost model and could be solution for settings where resources are limited.

It was evident that STEPS has made private sector accountable. The model is being owned by the private sector. Such accountability and ownerships were not seen in many other private sector engagement models [8]. We felt that it is a model by the private sector for ensuring standards of care to their patients by fostering customer loyalty. Fostering such ownerships could ensure sustainability. Given the huge presence of private sector in India, STEPS provide an opportunity for systematic and large-scale partnerships. Once the systems are established, dependency of private sector on NTEP could be minimal except for continuous supply of drugs and diagnostics. Private hospitals could even run STEPS as a profitable model without any support from NTEP or intermediary agency for day-to-day management and ensure standards of TB care to every patient. The only concern then would be the out-of-pocket expenditure incurred by the patient. Initiatives like IPAQT where TB tests are made available at affordable prices in the private sector could help in reducing out of pocket expenditure [18]. Patients or private hospitals getting reimbursed for drugs and diagnostics through health insurance schemes could be a long-term solution. Ayushman Bharat (National Health Protection Mission) in India gives tremendous opportunity to think in this direction [19].

This was the first formal evaluation of STEPS. The strengths of evaluation included (1) presence of a muti-disciplinary evaluation team (2) evaluation ascertaining inputs, processes and outcomes (3) validation of data from multiple sources including management information system, hospital records and qualitative interviewers with all stakeholders including patients and (4) attempt for a cost estimation from all perspectives. Major limitation of this evaluation is that, it was not designed to ascertain whether the increase in patient care indicators was due to STEPS alone. There was no major programmatic change in NTEP since 2018 that would have contributed to improvement in quality-of-care indicators. Sample size was determined arbitrarily considering the operational and logistic feasibility. Around 10% of all facilities which were selected through proper sampling were visited. Of the facilities visited, 60% were visited during January–March 2020 and the remaining were visited during November 2020–January 2021 due to the travel restrictions imposed by unprecedented situation of COVID-19. Though there was a time gap, we believe that it had not affected the quality of data collection. We relied on retrospective programmatic data for all cost driving activities and relied on qualitative insights of program managers, patients and private sector administrators wherever such data were not readily available. We did not collect patient cost in detail. We also did not include the impact of these pilots while calculating patient costs. We also have not calculated the potential cost saving due to the effectiveness of STEPS in averting future TB cases. Future comprehensive cost effectiveness study could augment our basic cost estimates.

## Conclusion

Evaluation confirmed that STEPS is a low cost and patient-centric strategy. STEPS also successfully addressed the gaps in the quality of care for patients seeking care in the private sector and ensured that services are aligned with the standards of TB care. STEPS could be scaled up to similar settings with appropriate contextual adaptations.

## Data Availability

The datasets used and/or analysed during the current study are available from the corresponding author on reasonable request.

## Abbreviations

AIC: Air borne Infection Control
COMPAcT: Coalition of Medical Professional Association against TB
IDI: In-depth Interview
JEET: Joint Efforts for Elimination of TB
NTEP: National TB Elimination Program
PHOTON: Private Hospitals’ Consortium
PPM: Public Private Mix
STEPS: System for TB Elimination in Private Sector
TB: Tuberculosis
UATBC: Universal Access to TB care
USD: US Dollar
